# Burnout and dropout associated with talent development in youth sports

**DOI:** 10.3389/fspor.2023.1190453

**Published:** 2023-06-02

**Authors:** André L. A. Soares, Humberto M. Carvalho

**Affiliations:** School of Sports, Federal University of Santa Catarina, Florianópolis, Santa Catarina, Brazil

**Keywords:** youth sports, specialization, injuries, evidence-based & research methodology, selection

## Introduction

Dropout and burnout are key issues in youth sports (1–5). However, evidence-based data on this topic is still scarce in literature, and it is important to be aware of its limitations before assuming any unique and conclusive interpretations about the development of young athletes through sports expertise.

Talent development programs in youth sports have been designed, structured, and financed by clubs and governmental bodies to promote conditions for young athletes to achieve high levels of performance, often as early as possible (6, 7). It is generally assumed that early engagement and accumulation of deliberate practice supervised by specialized coaches will improve the development of skills and competencies beyond the effects of normal growth and development needed to perform in high-level competitions (8–10). These programs are mostly based on systematic long-term specific training (e.g., deliberate practice) for athletes to perform in high-level competitions during adulthood (9, 11, 12). Inherent to this model, the attempt to predict potential talented athletes may occur at earlier ages, making the specialization in a single sport a consequent strategy in the practical field. It is assumed that the sooner individuals engage in focused practice, the larger will be the advantage in comparison with others (9, 13–15).

The main counterview to this approach is the Developmental Model of Sports Participation, which advocates participation in a range of sports with the purpose of fun and enjoyment, instead of focusing on dedication and skill acquisition in one sport (16). The model assumes that early specialization may be the reason for many negative issues related to the sports environment, such as an increasing risk of injuries, lack of enjoyment, negative psychosocial effects, and the occurrence of burnout or dropout (3, 16–18), even though the definition of specialization is often unclear (19–21).

The main purpose of this paper is to raise awareness of the limits of the available data and interpretations of dropout and burnout in youth sports, particularly considering the contexts of talent development.

## Participation and dropout in talent development

Commitment and engagement in highly demanding tasks, such as deliberate practice, require athletes to be highly motivated, and such accomplishment may determine athletes’ achievement in higher or lower levels of youth sports participation, or even lead to dropping out (8). The conceptualization of dropout is not standardized in the scientific literature, particularly considering the varying youth sports contexts studied, which limits comparisons of results and interpretations and guideline proposals. It seems reasonable to assume that dropout refers to youths who leave formal sports program participation. Patterns of progression in youth sports may vary according to the level of competition, participants’ age, commitment, achievement, and other external factors within sports participation. Due to the complexity and multifactorial influences in the selection process, young athletes may also have different opportunities and access to engage in specialized programs and better facilities throughout their career development as potential assets (22). For example, data in youth football highlights that talent identification and development is often biased by maturation-related differences in young athletes, which often results in an overrepresentation of early maturers in youth football (7, 23–25).

Several studies have suggested that early specialization is leading practitioners to exclude young athletes, leading to youth sports dropout and further evasion of practice (17, 26–28). Furthermore, the available data considering sports participation trends and dropout in youth sports considers varying levels of sport participation but is scarce when it comes to talent development contexts.

Recent reviews focused on the reasons for dropout in different contexts of youth sports suggested that influential factors leading to dropout were mostly related to psychological issues originating from athletes’ personal lives and social relationships, but no evidence indicated that early specialization was a predictor for burnout and dropout (29, 30). It has been noted that the perceived reasons for practitioners dropping out of sports participation were (a) having less support from their coaches, (b) more pressure to succeed in sports than others, and (c) fewer friends in the sport—mostly related to perception of competence and motivation. Athletes’ perception of competence may also be influenced by technical skills competence (24), related to their own perceptions as a consequence of the selection process along their career (29, 30). Another key aspect is the athletes’ accumulated experience, which brings to a higher perception of the context and influences the level of commitment young athletes have within sport practice (29, 30). All these factors may likely be directly related to other fields than sports in athletes’ lives, such as academic and professional perspectives and expectancies (31).

In order to avoid dropout in youth sports, it has been suggested that sports organizations should better understand the causes for athletes’ evasion within their contexts to promote adequate strategies, policies, and practical interventions (29). Youth sports programs could promote different levels of participation in both competitive and recreational levels, and practices should stimulate athletes to be focused on learning new skills and developing their abilities while creating good and respectful relationships (29). The available data and synthesis of data highlight that the context and environment athletes are engaged in influence their perceptions and psychological aspects–competitive levels; club or extracurricular programs; competitive or recreational purposes (3, 32–34).

Empirical evidence of selection processes in youth sports is still scarce and lacks more in-depth knowledge about determinants for athletes’ progression (or not) along their career. However, recent studies have found that more accumulated experience presented an advantage in youth athletes’ progression in their career (25, 32), and the performance in youth ages could influence professional level achievement (35). Satisfying psychological issues might promote a longer period athletes keep engaged in sports practice once the evasion seems to be caused by them. Thus, the assumption that early specialization leads to evasion may be unreliable according to the above-mentioned findings.

## Increased injury risk

Pathways to professionalization demand high amounts of training volume and increased loads. The selection process may be determinant for players’ achievement of higher levels of competition, and the main stakeholders make decisions based on subjective perspectives of observation of in-game performance (35, 36). Indeed, the risk of injuries may be a key issue and determine young players’ career continuity or not into professionalization. Due to the selection process of young players and the continuity of athletes’ careers, the risk of injuries is a critical issue. The consequences of injury occurrence may be determinant for athletes’ progression, dropout, or achievement of the competitive level. Consequently, a perceived increase in sport-related injuries has become another issue of research frequently assumed to be related to specialization (4, 14, 26, 37).

In general, current consensus statements assume that early specialization and intensive training in youth sports are related to high rates of injury (26, 28). However, interpretations are based on data with varying levels of youth sports exposure and participation, limiting the interpretations. Only recently, prospective data considering a heterogeneous sample of young athletes engaged in talent development contexts noted the contrasting high impacts on injuries and illnesses (38). The same research group noted that early specialization did not increase the risk of injury among young athletes in talent development programs (39). The data contrasts with general observations arguing that the occurrence of injury in specialized athletes, when compared to peers with contrasting youth sports levels (28). It seems reasonable to note that sports programs should adequately adjust the training exposure to athletes’ capacities and readiness into training volume and loads (38–40). In order to collaborate with practical intervention and sports programs, the challenge until now is to know the trends of youth development and the impact from multiple dimensions within athletes’ careers.

## Lack of enjoyment

Early specialization in a single sport has been hypothesized to lead athletes to a lack of enjoyment in sports practice due to the high monotony of routines and the high amount of time spent in specific training (3, 4, 41). Again, the available data considering the links between psychological characteristics and dropout is based on varying contexts of youth sports but is scarce when considering talent development settings.

Nevertheless, some recent findings have shown no substantial effects from the age of specialization on motivation and sources of enjoyment (20). Furthermore, our data (32) suggested that differences could be found according to the competitive level among female basketball players (32). Observations from our research, based on repeated measurements in female young basketball and volleyball players, showed a trend of association of enjoyment with chronological and biological age (distance to menarche), but no relation to accumulated exposure to sport-specific deliberate practice (21). Apparently, enjoyment is potentially linked to the environment athletes are engaged in and biological determinants more than the accumulated time of sport participation. In a sample of young swimmers aged 12–13 years, there were no associations between burnout and dropout with early specialization (4). Thus, different influences, such as coach-athlete relationship, social and parental support, relationships with peers, and alignment of achievement expectancies with personal, professional, and social lives dimensions merit analysis from a more comprehensive perspective (e.g., bioecological approach) to provide deeper insights into the links of dropout and enjoyment in talent development contexts.

## Current issues, challenges and future research lines in drop-out research in youth sports and talent development settings

Due to the multiple factors that influence athletes’ dropout from sports, research has not considered appropriate analytical approaches to considering not only intra- and inter-individual characteristics but also different structural factors, such as environmental, social, and political contexts in which sports programs are conducted (42). Other empirical and practical variables may be considered, including socio-demographical factors, diverse youth sport participation, age group ranges, and competitive levels athletes have experienced. To accomplish this task, hierarchical/multilevel frameworks should be considered as a default, as noted in other research areas (43, 44).

Research considering dropout in youth sports has been conducted through two different strategies: quantitative, by applying questionnaires related to possible factors associated with dropout on continued participation, and qualitative, through semi-structured interviews conducted to assess the factors and processes involved in dropping out of sports. Both strategies have been applied retrospectively. There is a need for prospective longitudinal designed investigations to describe a follow-up of youth athletes’ career progression (25, 29, 30, 32).

Another issue lies in the limitations when comparing different results in dropout studies. The conceptualization of dropout remains unstandardized; therefore, there may be different interpretations of the phenomenon. In general, studies considering dropout in youth sports have the following overlaps when considering dropout: (a) re-registration (or not) for a successive season in the sport context, (b) absent participation for two consecutive years (seasons), and (c) an extended period without practicing the sport. In this sense, it would be key that studies at least state the concept of dropout (4, 29, 30).

Given the highly selective population of youth athletes who aim to achieve professional levels and the boundaries to investigate them longitudinally, many studies have considered small populations and generalized their interpretations (a concern for replication in sports and exercise science (45)). Data collection may occur by convenience in specific clubs or programs, considering small samples of athletes who may or may not achieve higher levels of competition. Limitations and uniqueness of data setting in talent development settings should be assumed and discussed more often. Even considering retrospective findings about high-level athletes’ background convergences, there are limits to drawing causal interpretations for expertise attainment.

Prospective design studies, considering the data, context limitations, and multiple sources of information, with clear conceptual and theoretical frameworks, and combined with available advanced modeling approaches, may provide a path to advance the understanding of young athletes within talent development settings and its impacts on young athletes’ development.

## Conclusion

Most models and recommendations for youth athlete development have been designed to promote a better approach and guide coaches’ interventions throughout athletes’ career progress. Current consensus statements in youth sports highlight the potential links of sport specialization, particularly early specialization, with dropout and burnout in youth sports, and this is generalized to talent development contexts. However, it is clear that the body of evidence needs to overcome current limits in conceptual, methodological, and analytical approaches to provide better quality information to guide sports programs and coaches’ interventions in youth populations.
